# La prise en charge du pneumothorax spontané: à propos de 138 cas

**DOI:** 10.11604/pamj.2017.26.152.11437

**Published:** 2017-03-15

**Authors:** Bouchra Habibi, Leila Achachi, Sohaib Hayoun, Mohammed Raoufi, Laila Herrak, Mustapha El Ftouh

**Affiliations:** 1 Service de Pneumologie, CHU Ibn Sina, Rabat, Maroc

**Keywords:** Pneumothorax spontané, traitement, pathologie pleurale, drainage pleurale, Spontaneous pneumothorax, treatment, pleural pathology, pleural drainage

## Abstract

Le pneumothorax est définit par la présence d’air dans la cavité pleurale. L’objectif de notre étude rétrospective du pneumothorax spontanés au servie de pneumologie à l’hôpital Ibn Sina rabat (2009-2011) est de déterminer le profil épidémiologique, clinique, radiologique, thérapeutique et évolutif. Il s’agit de 138 patients: 128 hommes et 10 femmes (17 à 83 ans), un âge moyen de 44,5 +/- 17,4 ans; sexe ratio 12/8. Le tabagisme est noté chez 81,2%. La symptomatologie clinique est la douleur thoracique (92%), la dyspnée (60%). Et sur la radiographie thoracique: on trouve un PNO (pneumothorax) unilatéral total (110 cas); partiel (10 cas); localisé (6 cas); bilatéral (4 cas); à droite dans 51,4% et à gauche dans 45,7%. On a recensé 70% de pneumothorax spontanés primitifs et 30% de PNO secondaire à (BPCO 44%, et tuberculose pulmonaire 39%). La prise en charge initiale est l’hospitalisation de tous les patients : le drainage thoracique (95%), l’exsufflation à l’aiguille (1%). Le repos et l’O_2_ (4%). Le retour du poumon à la paroi a été obtenu avant 10 jours chez 63%. L’évolution est favorable chez 89%. Et les complications immédiates: l’emphysème sous cutané (5 cas); une infection (6 cas) et 3 décès (arrêt cardio-respiratoire); les complications à distance sont les récidives dans 11,6%; une 1^ère^ récidive chez 13 cas (drainage thoracique chez 11 cas et oxygénothérapie chez 2 cas) et une 2^ème^ récidive chez 3 cas (recours à la chirurgie). Ce travail montre l’intérêt du drainage thoracique et la surveillance dans la prise en charge du pneumothorax pour éviter les complications et surtout pour éviter les récidives avec un éventuel recours à la chirurgie.

## Abstract (en)

Pneumothorax is a collection of air in the pleural cavity. We conducted a retrospective study of patients with spontaneous pneumothorax in the Department of Pneumology at the Ibn Sina Hospital in Rabat (2009-2011) with the aim to determine the epidemiological, clinical, radiological, therapeutic and evolutionary manifestation of spontaneous pneumothorax. The study involved 138 patients: 128 men and 10 women (17-83 years), with an average age of 44.5 +/- 17.4 years and sex ratio of 12/8. 81.2% of patients were smokers. Clinical symptomatology was chest pain (92%), dyspnea (60%). Chest radiograph showed total unilateral (110 cases); partial (10 cases); localized (6 cases); bilateral (4 cases); right (51.4%) or left (45.7%) PNO (pneumothorax). During our study period we found that 70% of patients had spontaneous primitive pneumothorax and 30% had PNO secondary to Chronic obstructive pulmonary disease (COPD) (44%) and pulmonary tuberculosis (TB) (39%). Initial management included patients hospitalization, chest drainage (95%), needle exsufflation (1%), rest and O_2_ (4%). It enables the lung to stick to the chest wall within 10 days in 63% of patients. Evolution was favorable in 89% of patients. Immediate complications included: subcutaneous emphysema (5 cases); infection (6 cases) and 3 deaths (cardiorespiratory arrest). Late complications included: recurrences in 11.6%; the first recurrence occurred in 13 cases (chest drainage in 11 cases and oxygen therapy in 2 cases) while the second recurrence occurred in 3 cases (surgery). This study shows the role of chest drainage and monitoring in the management of pneumothorax to avoid complications and especially to prevent recurrences, with a possible need to resort to surgery.

## Introduction

Le pneumothorax est une urgence, affection fréquente en pathologie respiratoire et reste un problème de santé publique [1, 2]. Le plus souvent bien tolérée, mais pouvant engager le pronostic vital lorsqu’elle est compliquée [3]. Le pneumothorax spontané reste assez fréquent dans notre pays [4]. Il est défini par la présence d’air dans la cavité pleurale normalement virtuelle responsable d’un collapsus partiel ou compliquée [5]. C’est une cause classique et potentiellement grave de dyspnée aiguë ou de douleurs thoracique, chez les sujets vus aux urgences. Les signes cliniques et radiologiques de gravité doivent être systématiquement recherchés et surveillés [6]. L’analyse radio tomodensitométrique doit être un temps important du diagnostic positif et étiologique [7]. Le but du traitement du pneumothorax spontané sont d’obtenir une réexpansion pulmonaire complète, et traiter éventuellement la cause et de prévenir la récidive [8, 9]. Le but de ce travail est de déterminer le profil épidémiologique, clinique, radiologique, thérapeutique et évolutif des pneumothorax spontanés pris en charge au service de pneumologie de l’hôpital Ibn Sina de Rabat.

## Méthodes

Il s'agit d'une étude rétrospective ayant concerné 138 patients hospitalisés pour prise en charge d’un pneumothorax spontané au service de pneumologie à l’hôpital Ibn Sina sur une période allant de janvier 2009 à Décembre 2011. L’attitude thérapeutique pour la prise en charge des pneumothorax au service de pneumologie à l’hôpital Ibn Sina est la suivante [10]: en cas de pneumothorax unilatéral, partiel ou localisé, bien toléré: abstention avec repos au lit et oxygénothérapie; drainage thoracique si aggravation radiologique ou non retour du poumon à la paroi après 48 heures. Ce drain sera maintenu tant que le bullage persiste.

En cas de pneumothorax unilatéral, partiel ou localisé, mal toléré et pour un pneumothorax unilatéral total: drainage thoracique d’emblée. Pneumothorax bilatéral: drainage thoracique des deux côtés en commençant par le côté le moins décollé et en mettant en place une aiguille d’exsufflation du côté contro-latéral en attendant le drainage. Après la deuxième récidive, l’avivement pleural ou le talcage au cours d’une thoracoscopie en vue d’obtenir une symphyse pleurale est indiqué.

## Résultats

**Population à l'étude:** 138 pneumothorax ont été colligées durant cette période. L’âge moyen était de 44,5 +/- 17,4 ans, avec des extrêmes allant de 17 à 83 ans et un pic de fréquence entre 20 et 30 ans. On note une prédominance masculine dans 92,8% et 7,2% de femmes; avec un sexe ratio de 12,8.

**Antécédents:** Une intoxication tabagique a été retrouvée chez 81,2% des cas, et dont 42,8% sont sevrés et 37,7% sont toujours des fumeurs actifs. ATCDS de tuberculose pulmonaire chez 16 patients, 13,1% avaient un ATCDS de pneumothorax et 18 patients étaient porteurs d’une BPCO.

**Présentation clinique:** Le diagnostic positif de pneumothorax était posé sur l’examen clinique dont le maître symptôme est la douleur thoracique qui est notée dans 92% des cas suivi par la dyspnée dans 60% des cas.

**Les aspects radiologiques:** La radiographie thoracique permet de poser et confirmer le diagnostic du PNO dans 96% des patients ou il a objectivé un PNO unilatéral total chez 80% des cas, un PNO unilatéral partiel chez 7% des cas, un pneumothorax unilatéral localisé chez 4% des cas, un PNO bilatéral chez 3% des cas et 8 cas d’hydro-pneumothorax dont un cas s’est révèle un hémothorax au drainage, avec 51,4% de PNO droit contre 45,7% de PNO gauche.

**Les signes de gravités:** 78,3% des PNO étaient mal tolérés, avec présence de signes de gravité à type de polypnée dans 60% des cas et cyanose dans 47% des cas, tachycardie dans 30,4% et trouble de conscience chez un seul cas.

**Les étiologies du pneumothorax:** Les étiologies du PNO dépend de son type, le pneumothorax spontané primaire (dans 70% des cas), est survenu chez des patients sans pathologie pulmonaire sous-jacente connue alors que le pneumothorax spontané secondaire (30% des cas) est secondaire à la BPCO chez 44% des cas; à la tuberculose pulmonaire chez 39% des cas et secondaire à d’autres pathologies (fibrose pulmonaire; asthme et cancer pulmonaire) dans 17% des cas.

**La prise en charge thérapeutique du PNO:** La prise en charge initiale consistait en l’hospitalisation de tous les patients et l’attitude thérapeutique immédiate fut basée sur le type et la tolérance du pneumothorax: 131 patients (95%) ont bénéficié d’un drainage thoracique, soit devant l’existence d’un PNO total avec un décollement à l’apex supérieur à 3 cm et/ou supérieur à 2 cm en latéral ou devant un PNO partiel ou localisé mal toléré. L’exsufflation à l’aiguille a été préconisé chez un seul cas avec PNO unilatéral total mal toléré avec le recours au drainage après l’échec de l’exsufflation. Une abstention thérapeutique avec repos strict, oxygénothérapie et surveillance en milieu hospitalier ont été préconisés chez 4% des patients. L’évolution a été marquée par une aggravation clinique et/ou radiologique chez 3 patients ce qui a nécessité le recours au drainage thoracique.

**L’évolution:** > après le drainage thoracique; on a deux situations: l’arrêt du bullage avant ou à 10 jours a été obtenu chez 63% des patients drainés, avec retour du poumon à la paroi; une persistance du bullage au-delà de 10 jours a été notée chez 43 patients soit 31,2% des patients ayant un décollement du poumon à la paroi ce qui a nécessité soit un redrainage en cas de malposition du drain initial, soit la mise en place d’un deuxième drain avec bonne évolution. Pour 7 patients, devant la persistance du bullage, une thoracoscopie a été réalisée; soit par talcage, bullectomie ou thoracotomie. Le retour du poumon à la paroi a été obtenu en moyenne avant 10 jours dans 65% de ces patients. La durée du drainage et du redrainage variait de 10 à 28 jours.

> Après l’arrêt du bullage et le retour du poumon à la paroi, deux situations sont possibles : poumon radiologiquement sain: le drain est clampé 24 à 48 heures. Le drain est retiré s’il y’a pas de décollement du poumon après ce délai, dans notre contexte, la radiographie du thorax, après retour du poumon à la paroi, a objectivé un poumon radiologiquement sain dans 33%; poumon radiologiquement pathologique : a été retrouvé dans 67% des cas, le patient garde son drain pendant deux semaines en moyenne après l’arrêt du bullage.

**Les complications:** L’évolution est favorable chez 89% des cas.

**Les complications immédiates**: Etaient représentées par l’emphysème sous cutané dans 5% des cas ayant bien évolué, 3 cas de pleurésie purulente ayant nécessité une antibiothérapie et des aspirations quotidiennes avec bonne évolution. Il y avait eu 3 décès par arrêt cardio-respiratoire suite à un sepsis sévère.

**Les complications à distance du PNO**: Sont les récidives qui ont été survenues dans 11,6% des cas, avec une première récidive dans 9,4% des cas et une deuxième récidive dans 2,2%. On note un délai de 20 jours à > 10 ans. L’attitude thérapeutique après la première récidive est le drainage thoracique pour 11 cas et le repos au lit avec oxygénothérapie avec bonne évolution pour 2 cas. Après la survenue d’une deuxième récidive; les patients ayant nécessité un traitement chirurgical type thoracoscopie avec talcage ou bullectomie ou thoracotomie. Il n’y avait pas de récidive après la chirurgie.

## Discussion

Le pneumothorax spontané idiopathique est une pathologie relativement fréquente, avec la survenue en moyenne de 350 cas/an dans 30 services en Île de France selon les données de CUBREA de 1997 à 2001 [11]. En Angleterre, l’incidence du pneumothorax spontané idiopathique est de 26 cas pour 100 000 par an chez les hommes, et de 9 cas pour 100 000 par an chez les femmes [12]. Dans notre étude on a 138 cas durant une période de 3 ans à raison d’une moyenne de 46 cas par an. Le pneumothorax spontané primitif ou secondaire, touche essentiellement des sujets de sexe masculin. Sa survenue chez la femme est plus rare avec un sexe ratio estimé à 2,7/1 [13, 14]. Cette prédominance masculine est rapportée par de nombreuses études, ce qui rejoint notre étude avec un sexe ratio de 12,8. Dans notre série, la moyenne d’âge des patients est de 44,5 +/- 17,4 ans; ce qui rejoint les études rapportées par Deborah et Al [15]; Hiroshi et Al [16]. La prédominance masculine est expliquée par la fréquence élevée du tabagisme (facteur principal de la survenue du PNO pour les hommes par rapport aux femmes. Dans notre série, 81,9% des patients sont tabagiques; dans l’étude de l’équipe de l’hôpital Rouîba- Algérie, le tabagisme est retrouvé chez 72,5% des cas avec une consommation moyenne de 15,6 paquets/année [17]. Le lien entre le pneumothorax et le tabagisme surtout actif est étroit. Le diagnostic positif du pneumothorax repose essentiellement sur la clinique et la radiographie de thorax [18]. Dans notre étude, le tableau clinique est dominé par la douleur thoracique (92% des cas) et la dyspnée (60% des cas). Dans l’étude de l’équipe de l’hôpital de Blida-Algérie [19] le maitre symptôme est la douleur thoracique qui est notée dans 90% des cas suivi par la dyspnée dans 57,1% des cas. Dans celle de Fettal. N et Taleb. A [20] la symptomatologie est dominée par la douleur thoracique dans 56%. Les signes fonctionnels dans l’étude de l’équipe de CHU FB, Monastir, Tunisie [21] sont la douleur thoracique (91,1%) et la dyspnée (66,6%). Donc d’après les études, on remarque que les principaux signes révélateurs du pneumothorax sont la douleur thoracique et la dyspnée. Dans notre série; la radiographie thoracique permet de poser et confirmer le diagnostic du PNO dans 96% des patients. La radiographie thoracique objective dans toutes les études [21, 22] une fréquence élevée du PNO unilatéral total suivi du partiel, avec des rares cas du PNO bilatéral. Avec une prédominance du siège droit dans toutes les études rejoindront notre étude; mais il n’y a pas dans la littérature une explication de cette prédominance droite du PNO. Et il n y’a pas une relation statistiquement significative (p : 0,8) [23]. En cas de doute de diagnostic avec une bulle d’emphysème géante; la TDM thoracique a permis de confirmer le diagnostic du PNO chez 4% des patients dans notre étude. Dans notre étude le pourcentage du pneumothorax spontané primaire (70%) est élevé par rapport au pneumothorax spontané secondaire (30%); dans toutes les études les étiologies les plus fréquentes du PSS sont: la BPCO et la tuberculose. Sur le plan thérapeutique, tous les malades de notre série ont été hospitalisés et l’attitude thérapeutique immédiate fut basée sur le type et la tolérance du PNO. Dans notre étude, le choix du traitement est le drainage thoracique dans 94,4%; même choix pour l’équipe de Ruppet. A-M et ses collaborateurs [24] (80%); l’équipe de l’hôpital Abderrahmane; Tunisie [25] (84,6%) et dans l’étude EXPRED Desmettre T [26]. En France, il n’y a pas de consensus, les pneumologues ont une attitude partagée et les chirurgiens thoraciques sont majoritairement pratiquants du drainage en première intention [27]. Actuellement, le choix et l’utilisation de l’une ou l’autre technique dans cette indication reposant donc avant tout sur l’expérience et l’habitude des équipes prenant en charge ces patients, conduisant à proposer en première intention le drainage, ou parfois l’exsufflation. Le poids des habitudes laisse en retrait les recommandations proposées par des groupes d’experts internationaux (ACCP; BTS). Les principales critiques faites à ces recommandations concernent, la rareté des études randomisées, le caractère complémentaire, mais parfois divergent des définitions, la divergence des recommandations: drainage en première intention pour l’ACCP versus exsufflation à l’aiguille pour la BTS, et les questions laissées en suspens (aspiration versus drainage libre, place des valves antiretour et de la prise en charge ambulatoire…) [28]. L’évolution est favorable chez 89% des cas de notre étude; même résultat dans toutes les études. Dans notre étude le taux de récidives (11%) des cas est inférieur aux résultats de l’étude de Desmettre T. [29] avec un taux de récidives de 20%. En concluant que la récidive est présente quel que soit le traitement proposé, aspiration à l’aiguille ou drainage. Rabbat A. et ses collaborateurs [30] ont trouvé que les principaux facteurs de risque de récidive étaient représentés par le tabagisme et l’existence d’une pathologie pulmonaire préexistante dans ce cas.

## Conclusion

Le pneumothorax représente une pathologie fréquente en médecine d’urgence. Les lésions emphysémateuses participent à la physiopathologie des PSP est le tabagisme est clairement un facteur de risque. Il nécessite une recherche étiologique complète afin de classer le pneumothorax en spontané idiopathique ou en secondaire à une atteinte pulmonaire préexistante. L’analyse radio tomodensitométrique doit donc être un temps important du diagnostic, aussi bien positif qu’étiologique. Il convient de distinguer la prise en charge de première intention du PNO proprement dit (évacuation de l’épanchement pleural aérique), de la prévention des récidives. Les habitudes thérapeutiques régissent souvent la prise en charge du PNO. La variabilité des pratiques d’un centre hospitalier à un autre, voire d’un service à un autre est illustrée par le nombre de spécialistes sollicités et la diversité des techniques disponibles (simple observation, à l’oxygénothérapie, à l’aspiration à l’aiguille ou à l’aide d’un petit cathéter, au drainage thoracique, voire à la chirurgie). Le poids des habitudes laisse en retrait les recommandations proposées par des groupes d’experts internationaux (ACCP, BTS): drainage thoracique en première intention pour l’ACCP versus exsufflation à l’aiguille pour la BTS. Le sevrage tabagique est un impératif de prévention pour diminuer les risques de récidive. De même le sujet jeune est le plus souvent actif physiquement et peut représenter un facteur favorisant les récidives.

### Etat des connaissances actuelles sur le sujet

- Le pneumothorax représente une pathologie fréquente en médecine d’urgence, nécessitant une prise en charge de première intention (évacuation de l’épanchement aérique), et de la prévention des récidives;
- En général, le poids des habitudes laisse en retrait les recommandations proposées par des groupes d’experts internationaux (ACCP, BTS).

### Contribution de notre étude à la connaissance

- Cette étude met en lumière le profil épidémiologique, clinique, radiologique, thérapeutique et évolutif des pneumothorax spontanés;
- Par ailleurs, nous rapportons notre expérience pour améliorer sa prise en charge.
